# Intensive breast screening in *BRCA2* mutation carriers is associated with reduced breast cancer specific and all cause mortality

**DOI:** 10.1186/s13053-016-0048-3

**Published:** 2016-04-14

**Authors:** D. G. Evans, E. F. Harkness, A. Howell, M. Wilson, E. Hurley, M. M. Holmen, K. U. Tharmaratnam, A. I. Hagen, Y. Lim, A. J. Maxwell, P. Moller

**Affiliations:** Genesis Breast Cancer Prevention Centre and Nightingale Breast Screening Centre, University Hospital of South Manchester, Southmoor Road, Wythenshawe, Manchester, M23 9LT UK; Genomic Medicine, St Mary’s Hospital, Oxford Road, Manchester Academic Health Sciences Centre, University of Manchester Institute of Human Development, Central, Manchester Foundation Trust, Manchester, M13 9WL UK; Centre for Imaging Sciences, Institute of Population Health, University of Manchester, Oxford Road, Manchester, M13 9PL UK; Department of Radiology and Nuclear Medicine, Oslo University Hospital, Oslo, Norway; Department of Mathematics, University of Oslo, Blindern, Oslo, Norway; Department of Breast and Endocrine Surgery, Trondheim University Hospital, Trondheim, Norway; Department of Laboratory Medicine, Children’s and Women’s Health, Faculty of Medicine, Norwegian University of Science and Technology, Trondheim, Norway; Inherited Cancer Research Group, The Norwegian Radium Hospital, Department for Medical Genetics, Oslo University Hospital, Oslo, Norway; Department of Tumour Biology, Institute of Cancer Research, The Norwegian Radium Hospital, Oslo University Hospital, Oslo, Norway; Department of Human Medicine, Universität Witten/Herdecke, Witten, Germany

**Keywords:** Breast cancer, Mammography, MRI, *BRCA2*, Survival, Kaplan-meier

## Abstract

**Background:**

The addition of annual MRI screening to mammography has heightened optimism that intensive screening along with improved treatments may substantially improve life expectancy of women at high risk of breast cancer. However, survival data from *BRCA2* mutation carriers undergoing intensive combined breast screening are scarce.

**Methods:**

We have collated the results of screening with either annual mammography or mammography with MRI in female *BRCA2* mutation carriers in Manchester and Oslo and use a Manchester control group of *BRCA2* mutation carriers who had their first breast cancer diagnosed without intensive screening.

**Results:**

Eighty-seven *BRCA2* mutation carriers had undergone combined (*n* = 34) or mammography (*n* = 53) screening compared to 274 without such intensive screening. Ten year breast cancer specific survival was 100 % in the combined group (95 % CI 82.5–100 %) and 85.5 % (95 % CI 72.6–98.4 %) in the mammography group compared to 74.6 % (95 % CI 66.6–82.6 %) in the control group. Better survival was driven by lymph node status (negative in 67 % of screened vs 39 % of unscreened women; *p* < 0.001) and a significantly greater proportion of intensively screened women had invasive breast cancers <2 cm at diagnosis (74.6 % vs 50.4 %; *p* = 0.002).

**Conclusion:**

Intensive combined breast cancer screening with annual MRI and mammography appears to improve survival from breast cancer in *BRCA2* mutation carriers. Data from larger groups are required to confirm the effectiveness of combined screening in *BRCA2* carriers.

## Background

High penetrance inherited breast cancer is mainly caused by pathogenic mutations in the *BRCA1* and *BRCA2* genes. When these genes were identified, it soon became clear that breast cancer in women with pathogenic mutations in *BRCA1* had worse prognostic features compared with women carrying pathogenic *BRCA2* mutations who have tumours more reflective of breast cancer in the general population although still with a marginally increased level of high grade tumours [1–3]. Whilst strongly associated with triple negative breast cancer, most young patients with this breast cancer subtype do not carry pathogenic *BRCA1* mutations [4]. Additionally the vast majority of older women with familial breast cancers with good prognostic markers and good prognosis do not carry pathogenic *BRCA2* mutations [5]. The majority of families with smaller aggregations of breast cancer do not yet have demonstrable underlying genetic defects and the majority of carriers of pathogenic mutations in *BRCA1* and *BRCA2* do not have strong aggregation of breast cancer in their families [6]. Although caused by genes involved in homologous DNA repair, breast cancers caused by *BRCA1* and *BRCA2* mutations appear to be rather different diseases.

Initially, all carriers of pathogenic *BRCA1/2* mutations were advised to be mammographically screened from a young age [1]. However the prognosis for *BRCA1*-associated breast cancer remained serious despite early mammography surveillance [7]. Consequently. Magnetic Resonance Imaging (MRI) was advised to improve early diagnosis, and with resultant downstaging of tumours at diagnosis being demonstrated [8–12]. This lead to hope for improved survival [13] based on projection of observations of tumours in patients without demonstrated *BRCA1* mutations, assuming that their biology and response to treatment were similar. A validation of this hope based on empirical observed outcome of MRI screening in *BRCA1* carriers is, however, still lacking - besides a few reports indicating that it may not be the case [14, 15].

The prevalence of pathogenic *BRCA2* mutations in breast cancer cases is, however, less than for *BRCA1* in most of Western Europe and North America, which may be why reports on the outcome of early diagnosis with MRI in carriers of pathogenic *BRCA2* mutations are even sparser. This is presumed to be why many reports on the effects of early diagnosis on inherited breast cancer have combined *BRCA1* and *BRCA2* mutation carriers together to provide sufficient enough cases to arrive at a significant conclusion. However, by combining two biologically different groups of tumours, the average may not be true for individual patients. We previously reported that the outcome of early diagnosis with mammography and MRI for carriers of pathogenic *BRCA1* mutations [16] was not as good as was hoped for. We now report that the observed outcome of mammography and MRI in the carriers of pathogenic *BRCA2* mutations was better: the outcome in screened women carrying a pathogenic *BRCA2* variant was significantly superior to non-screened controls.

## Material and methods

Randomised control trials of screening in *BRCA2* mutation carriers are not feasible given the evidence that MRI screening has been shown to be effective at early detection with small more node negative cancers identified [8–12] and offering less than indicated in the current guidelines was considered unacceptable. We therefore assessed our prospective screening in *BRCA2* mutation carriers compared with an unscreened pragmatic control group. Women unaffected by breast cancer undergoing either annual mammography or combined annual mammography and MRI breast screening with pathogenic mutations in the *BRCA2* gene at time of breast cancer diagnosis or who later became identified from post-diagnosis testing were eligible for this study. Screening took place at the Genesis Prevention Centre in Manchester and in the regional hospitals in Norway where Oslo University Hospital served the majority of the mutation carriers between 1990 and 2014. Known *BRCA2* mutation carriers aged 30–50 years were offered annual mammography from 1996 (there are some prospective data with mammography from 1990 in women later found to be *BRCA2* mutation carriers) with the addition of MRI from 1997 (aged 30–50 years) with 12–18 monthly mammography after age 50 in the Manchester series. In the Norwegian series carriers were offered annual mammography combined with MRI from 25 to 70 years of age from 2001 onwards. Before MRI was available, and in cases where the *BRCA2* mutation was not detected until later, all *BRCA2* mutation carriers were subject to annual mammography without MRI in both Manchester and Norway [7]. All women were followed prospectively from breast cancer diagnosis. In Manchester age at last follow up or death was determined from hospital notes or the North West Cancer Intelligence Service (NWCIS) in October 2012 and NHS tracing in June 2014. Cause of death was established from NWCIS. In Norway, the outpatient genetic clinic in Oslo referred all patients for each single screening examination. Resultant screening reports and other outcomes, including follow-up after cancer diagnosis and causes of death were noted in the medical files. All prospectively detected cases had blood samples stored to be analysed later with updated methods if no mutation was detected initially. All women with prospective breast cancer were offered full *BRCA1/2* testing with sequencing and Multiple Ligation dependant Probe Amplification (MLPA). In Manchester, 22/302 (7.3 %) prospective breast cancers had not been tested, but there were only two deaths in the non-tested group. None of the Norwegian women with breast cancer were untested.

The pragmatic controls were diagnosed between 1996 and 2014 and were obtained from the Manchester Regional Genetic Register for *BRCA2*. Controls had only undergone population 3-yearly screening by mammography from 50 to 69 years of age or had not undergone radiological surveillance at all. Mutation testing was carried out after diagnosis, sometimes up to 10-years later. Follow-up from diagnosis to death or last known date living was as above. Survival curves were compared by Kaplan-Meier analysis.

## Results

Combining the published series from Manchester and Oslo increases the number of *BRCA2* mutation carriers diagnosed with breast cancer in a combined MRI/mammography programme from 20 to 34 when only women unaffected with breast cancer at entry are included [15, 16]. Similarly, the mammography group was expanded from 30 to 53 women from the UK report [16] (Table 1). There were 8 interval cancers in the mammography alone group and two in the combined group one of which was a 13 mm node negative invasive cancer found at risk reducing mastectomy. There were 274 carriers identified in the comparison group of which 260 had invasive breast cancer. There have been no deaths in the MRI group. Age at diagnosis ranged from 33 to 74 years (median 43) and there were 180 years of follow up (range 0.0-13.1; mean 5.3; median 4.0). In the mammography alone group, age at diagnosis ranged from 28 to 77 years (median 48) and there were 404 years of follow up (range 0.3–19.4; mean 7.6; median 6.7). There were six deaths in the mammography group, five from breast cancer and one from primary lung cancer. Median date at diagnosis was 04/2006 in the screened group with first cancer identified in 1993, although the first MRI detected cancer was in 2000. The controls were diagnosed aged 22–72 years (median 46.1) with 1525.one years of follow up (range 0–16.6; mean 5.56; median 4.7). Median date at diagnosis was 04/2003. There were 41 deaths: 37 from breast cancer, three from ovarian cancer, and one from heart disease.

**Table 1:** Combined MRI/mammography, mammography and unscreened *BRCA2* mutation carriers survival from diagnosis

	Number	10-year survival all causes (95 % CI)	Log Rank (Mantel-Cox) *P* value for overall survival compared to unscreened	10-year survival breast cancer (95 % CI)	Log Rank (Mantel-Cox) *P* value overall survival compared to unscreened
Unscreened	274	70.8 (62.2 to 79.4)		74.6 (66.6 to 82.6)	
MRI/mammography	34	100 %	0.026	100 %	0.035
	6 prevalent				
	26 incident				
	2 Interval				
Mammography	53	85.5 (72.6 to 98.4)	0.142	85.5 (72.6 to 98.4)	0.196
	9 prevalent				
	36 incident				
	8 Interval				
Any screening	87 (5 detected on mammography only in MRI group and 6 with both modalities)	89.5 (79.5 to 99.5)	0.017	89.5 (79.5 to 99.5)	0.029

**Fig. 1:**
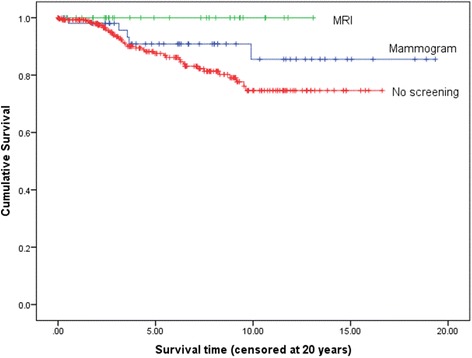
Breast cancer specific survival on Kaplan-Meier analysis for combined MRI and mammography versus no intensive screening

**Fig. 2:**
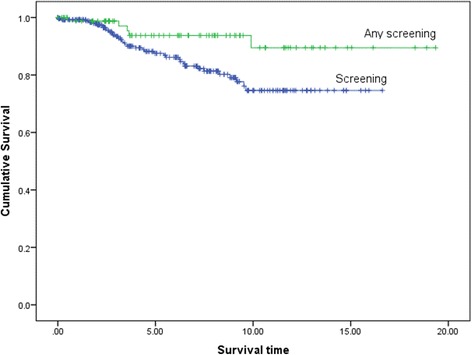
Kaplan-Meier survival plot for breast cancer specific deaths for those BRCA2 mutation carriers undergoing any intensive screening versus no additional screening (*p* = 0.029)

Ten-year overall and breast cancer specific survival was 100 % in the combined group and 85.5 % in the mammography group and 70.8 % and 74.6 % respectively in the controls (Table 1; Fig. 1). There were 16 alive without metastasis in the MRI group with more than 5-years follow up and nine with more than nine years follow up. Ten-year survival was 89.8 % in those *BRCA2* mutation carriers undergoing any form of intensive (mammography only or combined) screening compared with 74.6 % in controls (*p* = 0.026). Breast cancer specific survival remained significantly better at 20 years (Fig. 2).

Tumour characteristics are presented in Tables 2 and 3. Pathology was only available for grade on 175/260 (67 %) and for tumour size on 127/260 (49 %) of controls since many were diagnosed in other hospitals in the UK. However, the dates of diagnosis and whether they had invasive disease was known from cancer registration data for all controls. Tumours in the screened group were more likely to be DCIS: 19/87 (21.8 %) versus 14/274 in the comparison group (5.1 %) (*p* < 0.001). Invasive tumours in screened cases were significantly smaller (*p* = 0.002) and more likely to be node negative (Table 3: *p* = 0.001). The main driver of mortality appeared to be lymph node status; there was 83 % 10-year survival in node negative disease compared to 68 % for those with positive nodes (*p* = 0.019). Node negative screened cases did extremely well with 96 % 10-year survival compared to 72 % for node positive screened cases (*p* = 0.049). Surprisingly, tumour size and grade did not predict survival, although this may be confounded by the low proportion of unscreened women (among whom most of the deaths occurred) with full pathology data available. Age also appeared to have no effect in either the screened or unscreened groups.

**Table 2:** Tumour and age characteristics in intensively screened and unscreened women

			MRI/mammography	Mammography	No screening	Total	*p* value
In situ (*n* = 33, 79.1 %)		Ductal	10 (100)	9 (100)	14 (100)	33 (100)	1.0
		Lobular	0 (0)	0 (0)	0 (0)	0 (0)	
Invasive breast cancer (*n* = 328, 90.9 %)	Type (*n* = 328)	Ductal	24 (96.0)	41 (95.3)	244 (93.8)	309 (94.2)	
		Lobular	1 (4.0)	1 (2.3)	16 (6.2)	18 (5.5)	
		Mixed	0 (0)	1 (2.3)	0 (0)	1 (0.3)	
	Age groups (*n* = 328)	<50 years	17 (68.0)	23 (53.5)	168 (64.6)	208 (63.4)	0.330
		> = 50 Years	8 (32.0)	20 (46.5)	92 (35.4)	120 (36.6)	
	Nodes (*n* = 148)	0	18 (78.3)	23 (60.5)	34 (39.1)	75 (50.7)	
		1	2 (8.7)	8 (21.1)	17 (19.5)	27 (18.2)	
		2–3	1 (4.3)	6 (15.8)	17 (19.5)	24 (16.2)	
		4–5	0 (0)	1 (2.6)	9 (10.3)	10 (6.8)	
		6+	2 (8.7)	0 (0.0)	10 (11.5)	12 (8.1)	
		Missing	2	5	173	180	
	Nodes (*n* = 148)	Negative	18 (78.3)	23 (60.5)	34 (39.1)	75 (50.7)	0.001
		Positive	5 (21.7)	15 (39.5)	53 (60.9)	73 (49.3)	
	ER-status (*n* = 211)	Negative	3 (18.8)	11 (27.5)	34 (21.9)	48 (22.7)	0.698
		Positive	13 (81.3)	29 (72.5)	121 (78.1)	163 (77.3)	
		Missing	9	3	105	117	
	Grade (*n* = 237)	1	1 (4.3)	4 (10.3)	6 (3.4)	11 (4.6)	
		2	11 (47.8)	15 (38.5)	63 (36.0)	89 (37.6)	
		3	11 (47.8)	20 (51.3)	106 (60.6)	137 (57.8)	
		Missing	2	4	86	91	
	Grade (*n* = 237)	1/2	12 (52.2)	19 (48.7)	69 (39.4)	100 (42.2)	0.338
		3	11 (47.8)	20 (51.3)	106 (60.6)	137 (57.8)	
	Censored (*n* = 328)	Alive	25 (100)	38 (88.4)	222 (85.4)	285 (86.9)	0.112
		Dead	0 (0.0)	5 (11.6)	38 (14.6)	43 (13.1)	
	Size (*n* = 186)	<10	9 (42.9)	10 (26.3)	9 (7.1)	28 (15.1)	
		10 – 19.9	8 (38.1)	17 (44.7)	55 (43.3)	80 (43.0)	
		20 – 29.9	3 (14.3)	9 (23.7)	36 (28.3)	48 (25.8)	
		30 – 39.9	0 (0.0)	1 (2.6)	12 (9.4)	13 (7.0)	
		40 – 49.9	0 (0.0)	1 (2.6)	7 (5.5)	8 (4.3)	
		> = 50	1 (4.8)	0 (0.0)	8 (6.3)	9 (4.8)	
		Missing	4	5	133	142	
	Size (*n* = 186)	<20	17 (81.0)	27 (71.1)	64 (50.4)	108 (58.1)	0.006
		> = 20	4 (19.0)	11 (28.9)	63 (49.6)	78 (41.9)

**Table 3:** Invasive cancers by tumour characteristics with all cause survival

Selection	Number of cases	Number of deaths	5 years survival (SE)	10 years survival (SE)	*p* value for 2^nd^ category (e.g. <50 vs 50+)	*p* value for screened vs no screening for each category
No screening	260	38	0.871 (0.025)	0.728 (0.043)	0.089	
Screened	68	5	0.917 (0.040)	0.863 (0.065)		
All (*n* = 328)						
<50 year	208	25	0.895 (0.025)	0.795 (0.040)	0.117	
50+ yrs	120	18	0.853 (0.040)	0.647 (0.088)		
Screened						
<50 year	40	3	0.941 (0.041)	0.869 (0.079)	0.690	0.216
50+ yrs	28	2	0.867 (0.088)	0.867 (0.088)		0.214
No screening						
<50 year	168	22	0.883 (0.030)	0.777 (0.045)	0.121	
50+ yrs	92	16	0.847 (0.045)	0.570 (0.111)		
All (*n* = 211)						
ER neg	48	3	0.913 (0.048)	0.913 (0.048)	0.130	
ER pos	163	23	0.909 (0.028)	0.686 (0.064)		
Screened						
ER neg	14	0	1	1	0.146	0.238
ER pos	42	5	0.862 (0.065)	0.755 (0.116)		0.694
Not screened						
ER neg	34	3	0.875 (0.068)	0.875 (0.068)	0.375	
ER pos	121	18	0.925 (0.030)	0.665 (0.074)		
All (*n* = 237)						
Grade 1/2	100	12	0.892 (0.039)	0.740 (0.076)	0.943	
Grade 3	137	18	0.887 (0.032)	0.776 (0.050)		
Screened						
Grade 1/2	31	2	0.900 (0.067)	0.900 (0.067)	0.980	0.248
Grade 3	31	2	0.920 (0.055)	0.920 (0.055)		0.185
No screening						
Grade 1/2	69	10	0.889 (0.047)	0.668 (0.101)	0.958	
Grade 3	106	16	0.876 (0.039)	0.729 (0.062)		
All (*n* = 148)						
Node neg (0)	75	5	0.958 (0.029)	0.829 (0.076)	**0.019**	
Node pos (1+)	73	14	0.823 (0.054)	0.676 (0.077)		
Screened						
Node neg (0)	41	1	0.962 (0.038)	0.962 (0.038)	**0.049**	0.209
Node pos (1+)	20	4	0.821 (0.094)	0.718 (0.127)		0.706
No screening						
Node neg (0)	34	4	0.955 (0.044)	0.728 (0.123)	0.164	
Node pos (1+)	53	10	0.822 (0.067)	0.667 (0.089)		
All (*n* = 186)						
<20 mm	108	14	0.922 (0.029)	0.752 (0.058)	0.884	
20 + mm	78	10	0.865 (0.048)	0.778 (0.064)		
Screened						
<20 mm	44	4	0.909 (0.051)	0.818 (0.098)	0.624	0.441
20 + mm	15	1	0.917 (0.080)	0.917 (0.080)		0.277
No screening						
<20 mm	64	10	0.939 (0.034)	0.717 (0.086)	0.792	
20 + mm	63	9	0.849 (0.057)	0.731 (0.081)		

Bold type indicates statistically significant results

*BRCA2* status was established on average six years post breast cancer diagnosis in the controls, with a median time of 4.7 years. HER2 data was only available on a small proportion of women from each group as HER2 testing was not fully implemented until 2006.

However, only around 7 % of *BRCA2* carriers known in Manchester (12/175) are HER2 positive and only one of the screened cases was known to be HER2 positive.

## Discussion

Although there is evidence for a projected improvement in survival from annual mammography screening in familial breast cancer (from those largely at low risk of *BRCA1/2*) under 50 years of age [17, 18], this is the first time that a prospectively observed reasonably large series of *BRCA2* carriers has been shown to have an apparent survival advantage from annual screening. Recently a Dutch group showed no improvement in survival, based on only two deaths out of 18 *BRCA2* related breast cancers compared to three events in controls [19]. Nonetheless the same group reported that annual mammography screening beyond 60 years of age in *BRCA1/2* carriers is associated with a marked improvement in tumour stage at diagnosis, with 58 % diagnosed at stage two or above with usual two-yearly screening compared to only 21 % in the annual group [20]. Additionally, the interval cancer rate was doubled by extending screening to two years. The data from this and the present study concur with NICE guidelines in England and Wales who recommend annual mammography for *BRCA1/2* carriers until 70 years of age [21]. Although the present study has used a pragmatic comparison group of *BRCA2* carriers not undergoing intensive screening a true matched control series would be impossible as women who knew they were mutation carriers would be very unlikely to not undergo added surveillance.

The current situation is that no single centre has a series large enough and well enough constructed and documented to provide a definitive answer to the question of whether MRI breast screening improves survival in *BRCA2* mutation carriers. This is why close to all major organisations world-wide addressing these questions have organised ‘THE BRCA CHALLENGE’ (http://www.humanvariomeproject.org/brca-challenge.html) which at the 2015 meeting in the UNESCO centre in Paris called for a broad international collaboration to provide answers to the unanswered questions. In this context we report our findings and encourage others to do the same, so as to move our knowledge on effects of interventions to prevent *BRCA2*-associated breast cancer death from assumptions to empirical observed effects of interventions. Until the time when more definitive answers are available female *BRCA2* carriers will still require guidance on whether surveillance with MRI and mammography offers similar improvements in life expectancy than can be gained from risk reducing surgery [13, 22].

## Abbreviations

CI: confidence interval
DCIS: ductal carcinoma in situ
DNA: BRCA1, BRCA2, MRI, magnetic resonance imaging
NHS: national health service
NICE: national institute for health and care excellence
NWCIS: north west cancer intelligence service

## References

[1] Moller P, Evans DG, Reis MM, Gregory H, Anderson E, Maehle L (2007). Surveillance for familial breast cancer: Differences in outcome according to BRCA mutation status. Int J Cancer.

[2] Lakhani SR, Reis-Filho JS, Fulford L, Penault-Llorca F, van der Vijver M, Parry S (2005). Prediction of BRCA1 status in patients with breast cancer using estrogen receptor and basal phenotype. Clin Cancer Res.

[3] Lakhani SR, Jacquemier J, Sloane JP, Gusterson BA, Anderson TJ, van de Vijver MJ (1998). Multifactorial analysis of differences between sporadic breast cancers and cancers involving BRCA1 and BRCA2 mutations. J Natl Cancer Inst.

[4] Evans DG, Howell A, Ward D, Lalloo F, Jones JL, Eccles DM (2011). Prevalence of BRCA1 and BRCA2 mutations in triple negative breast cancer. J Med Genet.

[5] Møller P, Stormorken A, Holmen MM, Hagen AI, Vabø A, Mæhle L (2014). The clinical utility of genetic testing in breast cancer kindreds: a prospective study in families without a demonstrable BRCA mutation. Breast Cancer Res Treat.

[6] Møller P, Hagen AI, Apold J, Maehle L, Clark N, Fiane B (2007). Genetic epidemiology of BRCA mutations--family history detects less than 50 % of the mutation carriers. Eur J Cancer.

[7] Møller P, Evans G, Haites N, Vasen H, Reis MM, Anderson E (1999). Guidelines for follow-up of women at high risk for inherited breast cancer: consensus statement from the Biomed 2 Demonstration Programme on Inherited Breast Cancer. Dis Markers.

[8] Kuhl CK, Schrading S, Leutner CC, Morakkabati-Spitz N, Wardelmann E, Fimmers R (2005). Mammography, breast ultrasound, and magnetic resonance imaging for surveillance of women at high familial risk for breast cancer. J Clin Oncol.

[9] Warner E, Plewes DB, Hill KA, Causer PA, Zubovits JT, Jong RA (2004). Surveillance of BRCA1 and BRCA2 mutation carriers with magnetic resonance imaging, ultrasound, mammography, and clinical breast examination. JAMA.

[10] MARIBS study group (2005). Screening with magnetic resonance imaging and mammography of a UK population at high familial risk of breast cancer: A prospective multicentre cohort study (MARIBS). Lancet.

[11] Sardanelli F, Podo F, D’Agnolo G, Verdecchia A, Santaquilani M, Musumeci R (2007). Multicenter comparative multimodality surveillance of women at genetic-familial high risk for breast cancer (HIBCRIT Study): interim results. Radiology.

[12] Hagen AI, Kvistad KA, Maehle L, Holmen MM, Aase H, Styr B (2007). Sensitivity of MRI versus conventional screening in diagnosis of BRCA-associated breast cancer in a national prospective series. Breast.

[13] Kurian AW, Sigal BM, Plevritis SK (2010). Survival analysis of cancer risk reduction strategies for BRCA1/2 mutation carriers. J Clin Oncol.

[14] Moller P, Stormorken A, Jonsrud C, Holmen MM, Hagen AI, Clark N (2013). Survival of patients with BRCA1-associated breast cancer diagnosed in an MRI-based surveillance program. Breast Cancer Res Treat.

[15] Evans DG, Kesavan N, Lim Y, Gadde S, Hurley E, Massat NJ (2014). MRI breast screening in high-risk women: cancer detection and survival analysis. Breast Cancer Res Treat.

[16] Møller P, Tharmaratnam K, Howell A, Stavrinos P, Sampson S, Wallace A (2015). Tumour characteristics and survival in familial breast cancer prospectively diagnosed by annual mammography. Breast Cancer Res Treat.

[17] FH01 collaborative teams (2010). Mammographic surveillance in women younger than 50 years who have a family history of breast cancer: tumor characteristics and projected effect on mortality in the prospective, single-arm, FH01 study. Lancet Oncol.

[18] Maurice A, Evans DG, Affen J, Greenhalgh R, Duffy SW, Howell A (2012). Surveillance of women at increased risk of breast cancer using mammography and clinical breast examination: Further evidence of benefit. Int J Cancer.

[19] Saadatmand S, Obdeijn IM, Rutgers EJ, Oosterwijk JC, Tollenaar RA, Woldringh GH, et al. Survival benefit in women with BRCA1 mutation or familial risk in the MRI screening study (MRISC). Int J Cancer 2015 Mar 26. doi:10.1002/ijc.29534 [Epub ahead of print]. 10.1002/ijc.29534 25820931

[20] Saadatmand S, Vos JR, Hooning MJ, Oosterwijk JC, Koppert LB, de Bock GH (2014). Hereditary Breast and Ovarian Cancer Research Group Netherlands (HEBON). Relevance and efficacy of breast cancer screening in BRCA1 and BRCA2 mutation carriers above 60 years: a national cohort study. Int J Cancer.

[21] McIntosh A, Shaw C, Evans G, Turnbull N, Bahar N, Barclay M, et al. (2004 updated 2006, 2013) Clinical Guidelines and Evidence Review for The Classification and Care of Women at Risk of Familial Breast Cancer, London: National Collaborating Centre for Primary Care/University of Sheffield. NICE guideline http://www.nice.org.uk/guidance/cg164.

[22] Ingham SL, Sperrin M, Baildam A, Ross GL, Clayton R, Lalloo F (2013). Risk-reducing surgery increases survival in BRCA1/2 mutation carriers unaffected at time of family referral. Breast Cancer Res Treat.

